# Supplementary material for: Factors that determine dependence in daily activities: A cross-sectional study of family practice non-attenders from Slovenia
Source: PLoS One. 2021 Jan 22;16(1):e0245465. doi: 10.1371/journal.pone.0245465 (PMC7822267; doi:10.1371/journal.pone.0245465)
Supplement: S1 Table — Note that there were no missing values with regard to the outcome or demographic characteristics. (DOCX) [file pone.0245465.s001.docx]

**S1 Table.** Comparison of the outcome (dependence) and demographic characteristics between the subsample used in the multivariate model for dependence in daily activities (n=1814) and the subsample that was omitted from the model on the account of missing values for the covariates in the model (n=185). Note that there were no missing values regarding the outcome or demographic characteristics.

| **Characteristic** | **Subsample used in the model**  (n=1814) | **Subsample omitted from the model**  (n=185) |
| --- | --- | --- |
| **Dependent**, n (%) | 423 (23.3) | 43 (23.2) |
| **Gender: female**, n (%) | 1153 (63.6) | 103 (55.7) |
| **Age** (years), median (IQR) | 61.5 (42.0 – 76.2) | 55.6 (39.7 – 73.2) |
